# Large hepatocellular carcinoma in a non-cirrhotic liver with peritoneal and omental metastasis in a healthy man: a case report

**DOI:** 10.1186/s13256-017-1203-9

**Published:** 2017-02-08

**Authors:** H. M. M. T. B. Herath, Aruna Kulatunga

**Affiliations:** National Hospital, Colombo, Sri Lanka

**Keywords:** Hepatocellular carcinoma, Intra-abdominal metastasis, Non-cirrhotic liver, Young male

## Abstract

**Background:**

Liver cancer is the second leading cause of cancer death in men worldwide. Hepatocellular carcinoma usually develops in the setting of cirrhosis or chronic inflammation. Major risk factors for developing hepatocellular carcinoma are chronic hepatitis B or C virus infection, alcoholic cirrhosis, and nonalcoholic fatty liver disease. The most frequent locations for hepatocellular carcinoma to metastasize are the lungs, portal vein, bones, and regional lymph nodes.

**Case presentation:**

A 41-year-old Sri Lankan man presented with progressive abdominal distension and on examination was found to have a palpable irregular mass in the left lobe of his liver with moderate ascites. His ascitic fluid was an exudate without malignant cells. An ultrasound scan and contrast-enhanced computed tomography of his abdomen showed a large contrast-enhancing lesion in the left lobe of his liver without features of cirrhosis. Laparoscopic assessment revealed peritoneal and omental deposits. Histology of the biopsies taken from the liver lesion, omental deposits, and peritoneal deposits supported a diagnosis of hepatocellular carcinoma. His liver biochemistry was normal and hepatitis serology was negative. He is abstinent from alcohol and did not have metabolic syndrome.

**Conclusions:**

It is rare for a young patient to develop hepatocellular carcinoma with a normal liver without chronic hepatitis B or C infection, or any other risk factors. Intraperitoneal metastasis of non-ruptured hepatocellular carcinoma is also very rare. Here we report a rare case of a 41-year-old man with a large hepatocellular carcinoma in a non-cirrhotic liver without chronic hepatitis who presented with peritoneal and omental metastasis.

## Background

Liver cancer is commoner in men than in women. In men, it is the second most leading cause of cancer death in less developed countries; in more developed countries, it is the sixth leading cause of cancer deaths among men [1]. The major risk factors for developing hepatocellular carcinoma (HCC) are hepatitis B viral infection, chronic hepatitis C virus (HCV) infection, alcoholic cirrhosis, hereditary hemochromatosis, nonalcoholic fatty liver disease, environmental toxins (aflatoxin and contaminated drinking water), and cirrhosis of almost any cause [2]. HCC in a non-cirrhotic liver without any of the risk factors is rare [3]. Intraperitoneal metastasis of a non-ruptured HCC is also rare [4]. Here we describe a 41-year-old man presenting with a large HCC in a non-cirrhotic liver with peritoneal and omental metastasis and moderate ascites. He was tested and was negative for hepatitis serology and other risk factors for cirrhosis. He refused any intervention and wished to be managed conservatively.

## Case presentation

A 41-year-old Sri Lankan man presented to our unit with progressive abdominal distension and discomfort for 3 months. He also noticed loss of appetite and loss of weight for the same period of time. There was no history of sudden epigastric or right hypochondrial pain, hematemesis, melena, per rectal bleeding, or altered bowel habits. Nor did he have any confusion or altered sleep pattern suggestive of hepatic encephalopathy. He was previously well with no prior diagnosis of diabetes mellitus, hypertension, or dyslipidemia. He was abstinent from alcohol and he was not a tobacco smoker; he denied any intravenous drug abuse, blood transfusions, sexual promiscuity, or past history of hepatitis. There was no family history of liver diseases or HCC.

On examination, he was not icteric or pale and there was no bilateral leg edema or peripheral stigmata of chronic liver cell disease. His cardiovascular system examination was normal with a normal blood pressure. His abdomen was distended with a palpable irregular mass in the left lobe of his liver without a bruit. There was no splenomegaly. A moderate amount of free fluid was present. A neurological examination revealed no signs of hepatic encephalopathy.

Initial basic investigations showed anemia and leukocytosis with normal liver and renal biochemistry (Table 1). His blood picture showed normochromic normocytic anemia with mild to moderate rouleaux formation and mild neutrophilic leukocytosis. His inflammatory markers were high with an erythrocyte sedimentation rate (ESR) of 125 in the first hour and a C-reactive protein (CRP) of 155 mg/L (<6). His urine full report was normal. His fasting blood sugar (5.4 mmol/L) and lipid profile were normal. His lactate dehydrogenase (LDH) was 669 U/L (230 to 460) and amylase was 68 U/L (22 to 80).

**Table 1:** Full blood count, liver function tests, renal function tests, and serum iron studies

Investigation	Normal range	Investigation	Normal range
WBC = 11.15 × 10^3^/μL	4–10 × 10^3^/μL	Neutrophil = 85.8%	50–70%
Lymphocyte = 8.4%	20–40%	Eosinophil = 0.03%	0.5–05%
Hemoglobin = 9.7 g/dL	11–16 g/dL	MCV = 92.4 fL	80–100 fL
MCH = 29.4 pg	27–34 pg	MCHC 31.8 g/dL	32–36 g/dL
RDW-CV 0.119	0.110–0.160	Serum creatinine = 1.13 mg/dL	0.68–1.36 mg/dL
Serum sodium = 140 mmol/L	135–145 mmol/L	Serum potassium = 4.7 mmol/L	3.5–5.1 mmol/L
Albumin = 37 g/L	36–50 g/L	Globulin = 41.0 g/L	22–40 g/L
Alkaline phosphatase = 84 U/L	30–120 U/L	GGT = 60 U/L (<55)	<55 U/L
ALT = 37 U/L	<50 U/L	AST = 26 U/L	<50 U/L
Total bilirubin = 6.7 μmol/L	5–21 μmol/L	INR = 1.2	<1.4
Serum magnesium = 1.1 mmol/L	0.8–1.1 mmol/L	Ionized calcium 1.27 mmol/L	1.0–1.3
Serum iron = 100.9 μg/dL	59–156 μg/dL	TIBC = 349	291–430
Transferrin saturation = 30.8%	20–50%	Ferritin = 388 ng/mL	28–365 ng/mL

*ALT* alanine aminotransferase *AST* aspartate aminotransferase *GGT* gamma-glutamyltransferase *INR* international normalized ratio *MCH* mean corpuscular hemoglobin *MCHC* mean corpuscular hemoglobin concentration *MCV* mean corpuscular volume *RDW* random distribution of red cell width *TIBC* total iron-binding capacity *WBC* White blood cell count.

Hepatitis B serology (hepatitis B surface antigen, hepatitis B surface antibody, and hepatitis B core antibody) and hepatitis C serology (HCV antibody and hepatitis C ribonucleic acid (RNA)) were negative as were human immunodeficiency virus (HIV) serology and Venereal Disease Research Laboratory (VDRL) test. Serum iron studies were within normal range except for marginally elevated serum ferritin (Table 1). His serum alpha-fetoprotein was 12 μg/L. The result of an antinuclear antibodies (ANA) test was negative and the ceruloplasmin level in the serum was normal.

**Fig. 1:**
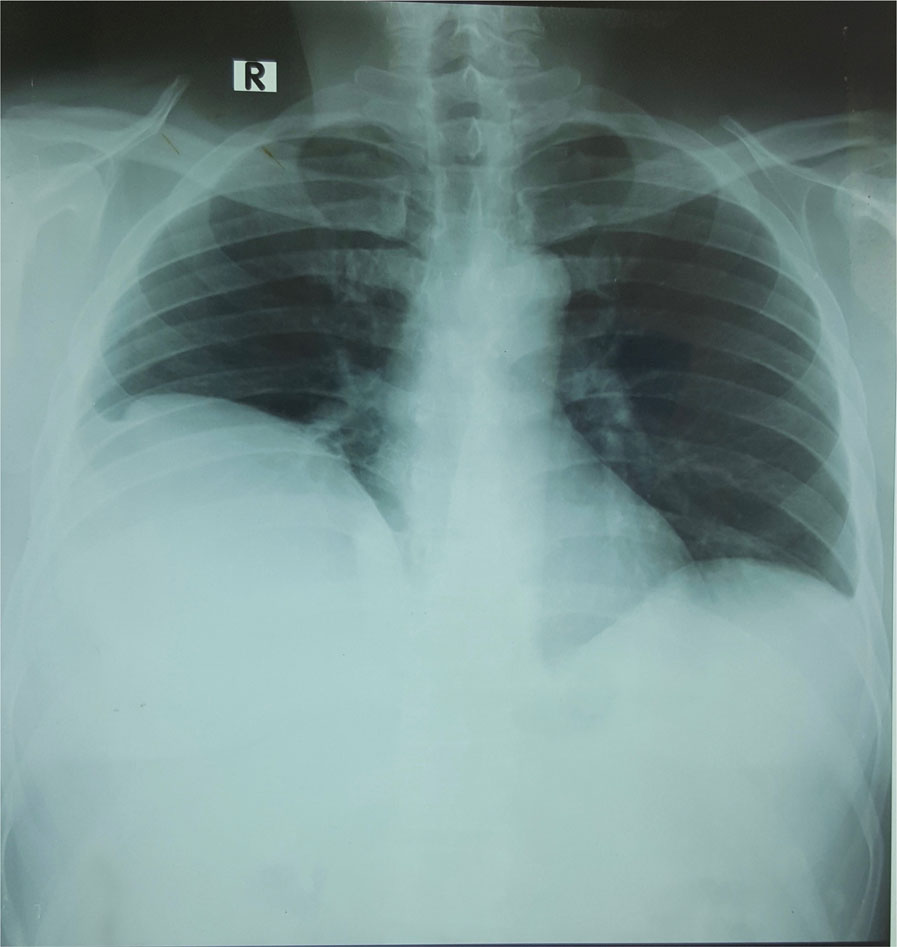
A posteroanterior chest X-ray showed right-side hemidiaphragm elevation

An ultrasound (USS) scan of his abdomen showed a heterogeneous ill-defined area of 4.5 × 2.4 cm in the left lobe of his liver with irregular margins. The rest of his liver was normal with normal outline and uniform echogenicity without features of cirrhosis. His gall bladder and bile ducts were normal. His spleen was mildly enlarged (12.6 cm) and moderate ascites was present. A portal vein Doppler showed normal flow pattern with no evidence of portal vein thrombosis (portal vein diameter 0.98 cm and flow velocity 25 cm/second). There was no para-aortic lymphadenopathy. His pancreas, kidneys, and prostate were normal. A posteroanterior chest X-ray (CXR-PA) showed elevation of his right hemidiaphragm (Fig. 1).

**Fig. 2:**
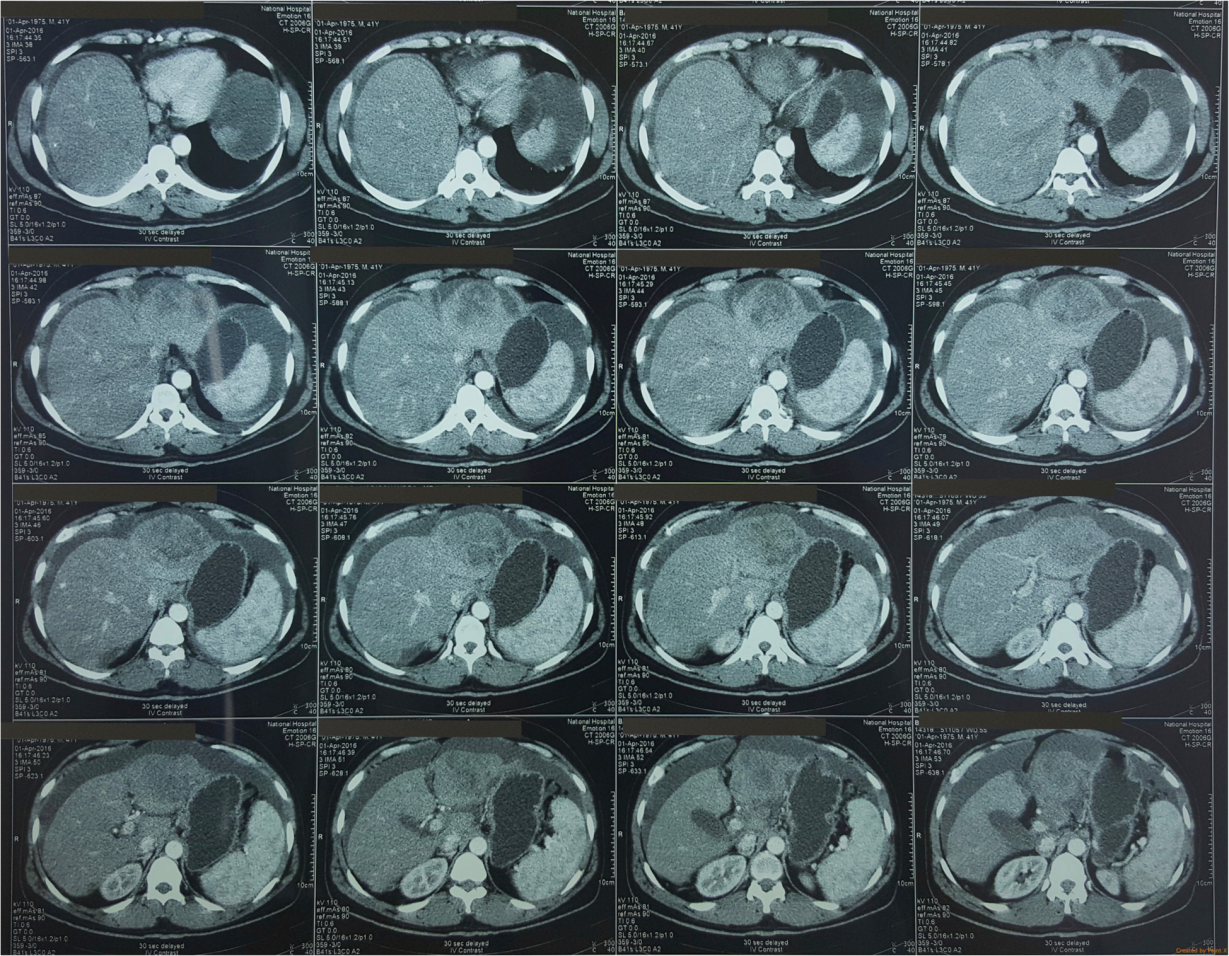
Liver lesion seen in 30 seconds, delayed film. Contrast-enhanced computed tomography of the abdomen

**Fig. 3:**
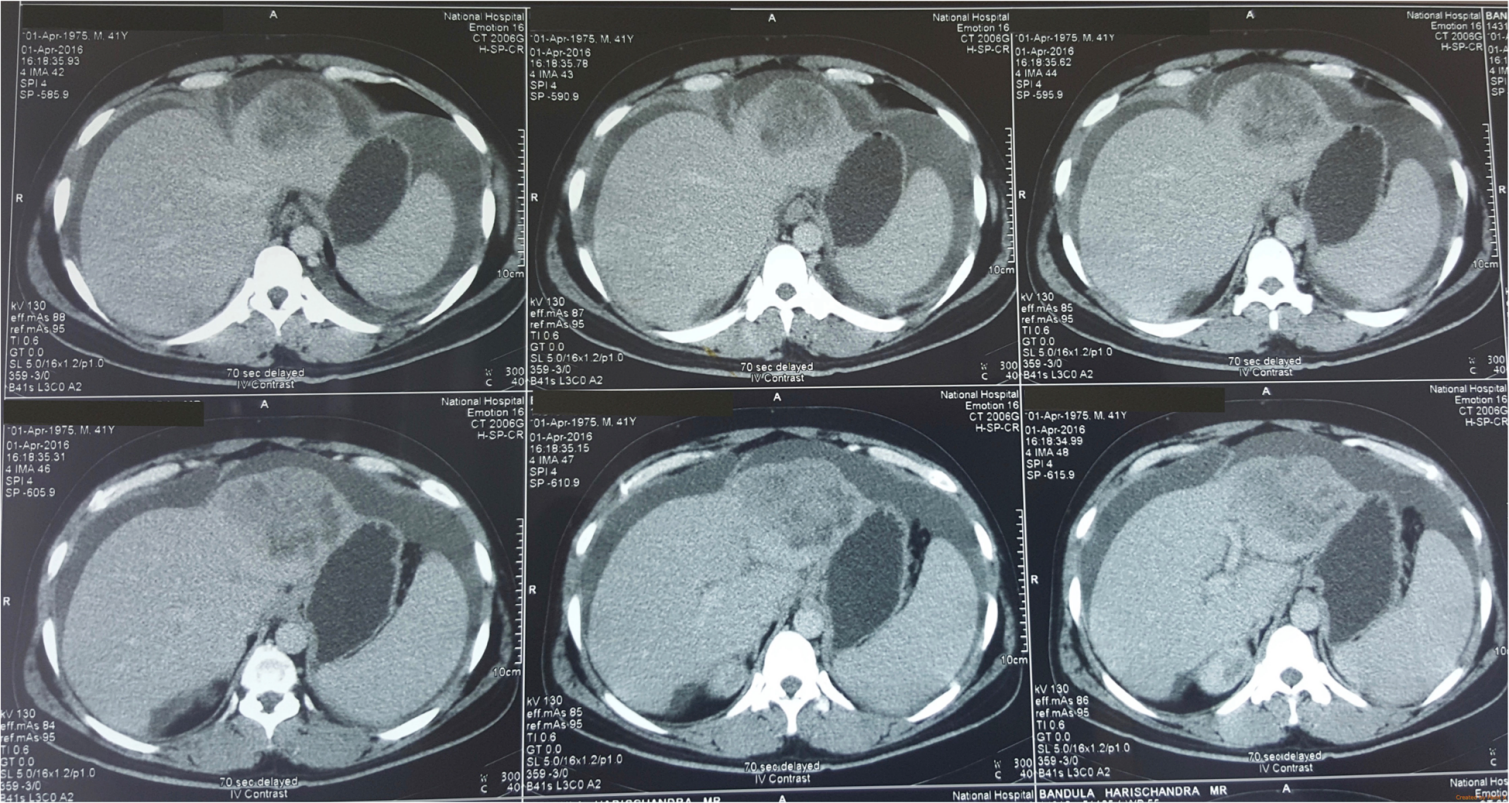
Liver lesion seen in 70 seconds, delayed film. Contrast-enhanced computed tomography of the abdomen

**Fig. 4:**
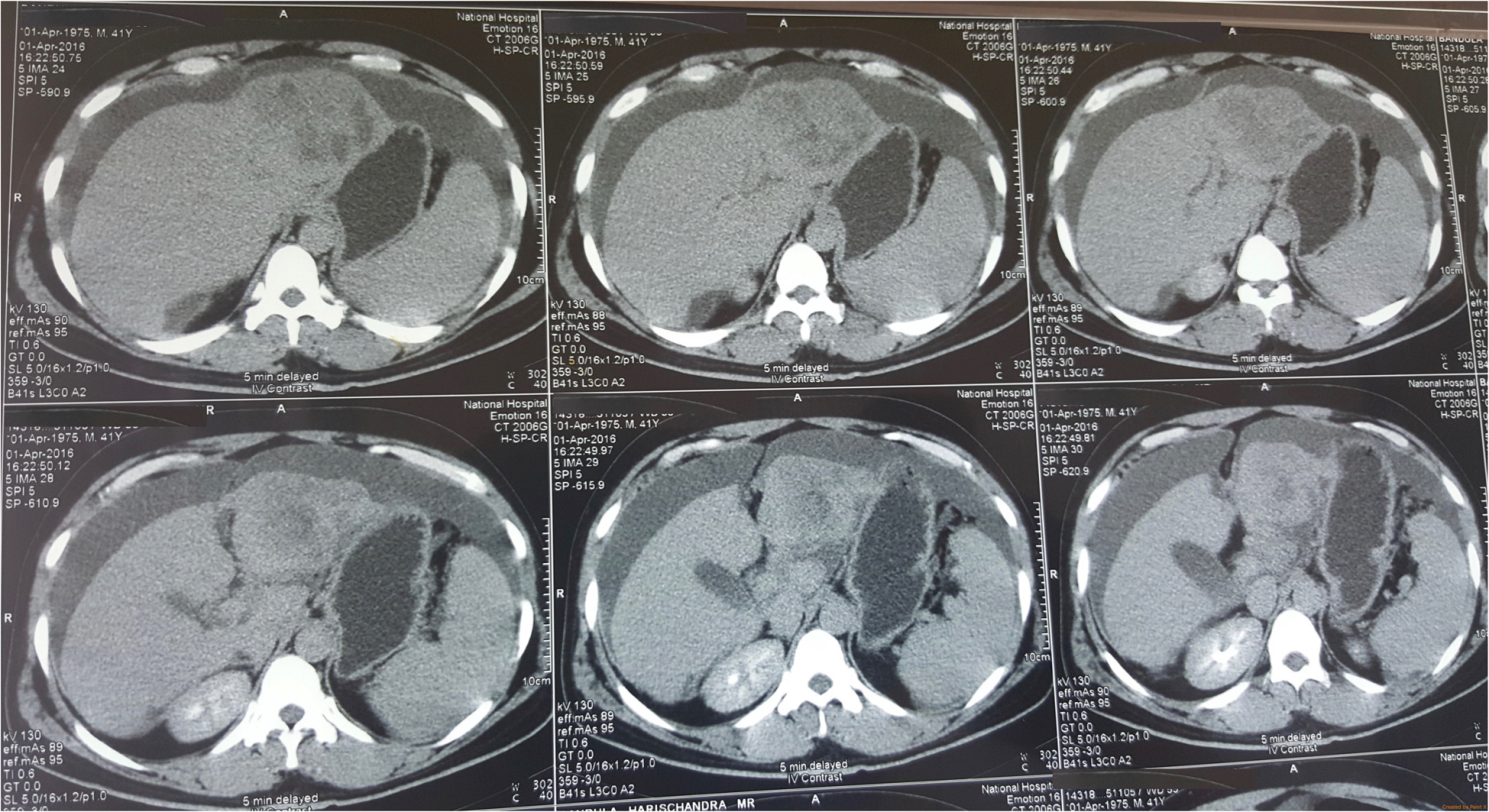
Liver lesion seen in 5 minutes, delayed film. Contrast-enhanced computed tomography of the abdomen

**Fig. 5:**
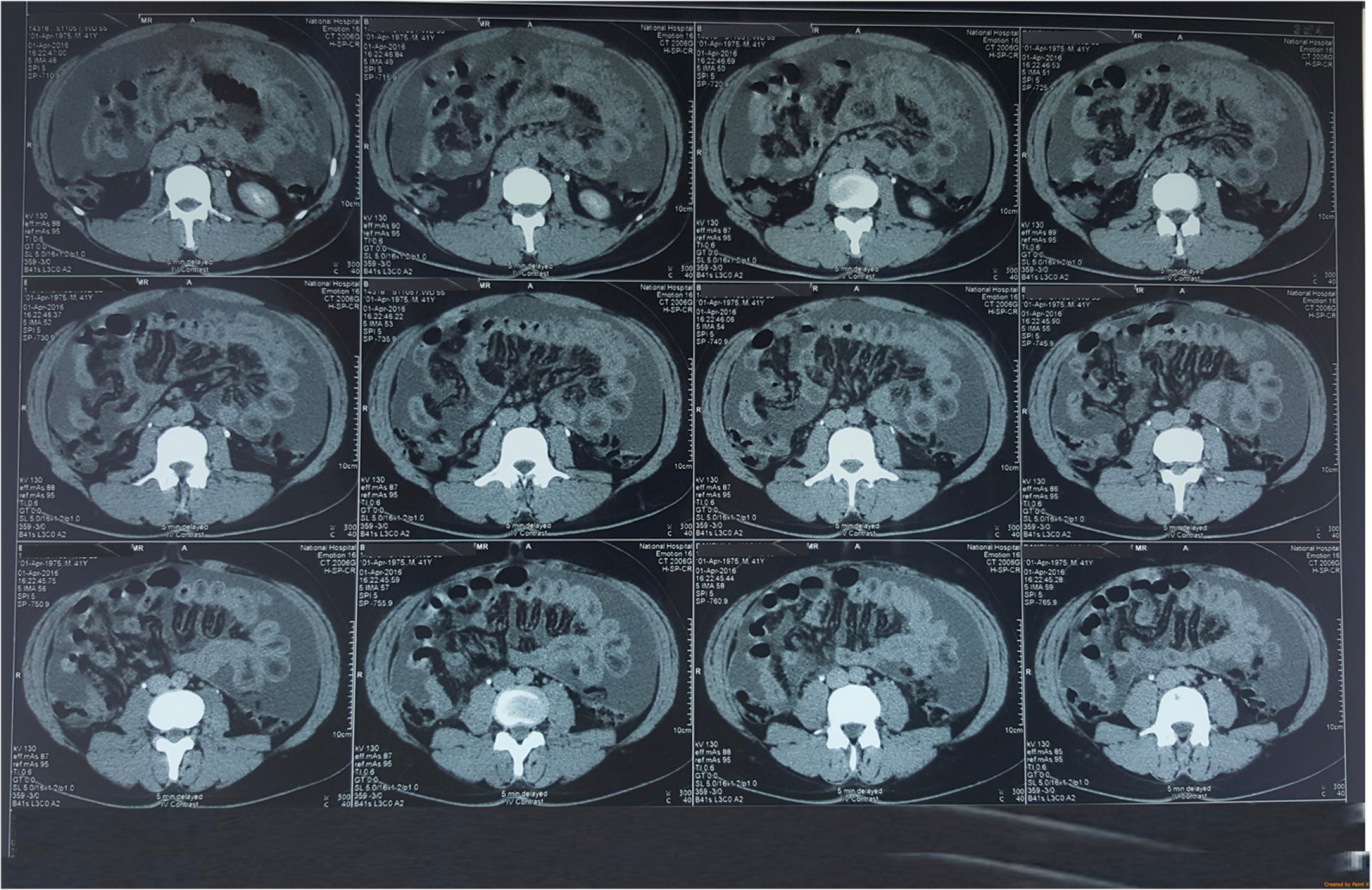
Gross ascites. Contrast-enhanced computed tomography of the abdomen

Contrast-enhanced triple-phase computed tomography (CT) showed a 7.5 × 6.5 cm lesion in the left lobe of his liver which enhanced during the arterial phase and showed a washout effect in the venous phase (Fig. 2 shows liver lesion seen at 30 seconds from contrast administration, delayed film; Fig. 3 shows liver lesion seen at 70 seconds, delayed film; and Fig. 4 shows liver lesion seen in 5 minutes, delayed film). The right lobe of his liver was normal. Intrahepatic and extrahepatic ducts were normal. There were no para-aortic masses. Gross ascites was evident (Fig. 5).

A diagnostic peritoneal tap revealed the ascitic fluid to be slightly turbid and mildly blood stained. His ascitic fluid albumin was 40 g/L (serum-to-ascites albumin gradient = -03 g/L) and it contained 25 neutrophils/mm^3^, 160 lymphocytes/mm^3^, and 4000 red blood cells/mm^3^. His glucose concentration was 4.0 mmol/L: random blood sugar (RBS) 7.0 mmol/l. His adenosine deaminase level was 29 U/L: tuberculosis (TB) 92 +/– 45, non-TB 12 +/– 11. An analysis of 200 mL of ascitic fluid for cytology showed reactive mesothelial cells, histiocytes, and lymphocytes. There was no evidence of atypical cells. A Gram stain and bacterial cultures of ascitic fluid were negative. Polymerase chain reaction (PCR) and culture for TB were also negative.

**Fig. 6:**
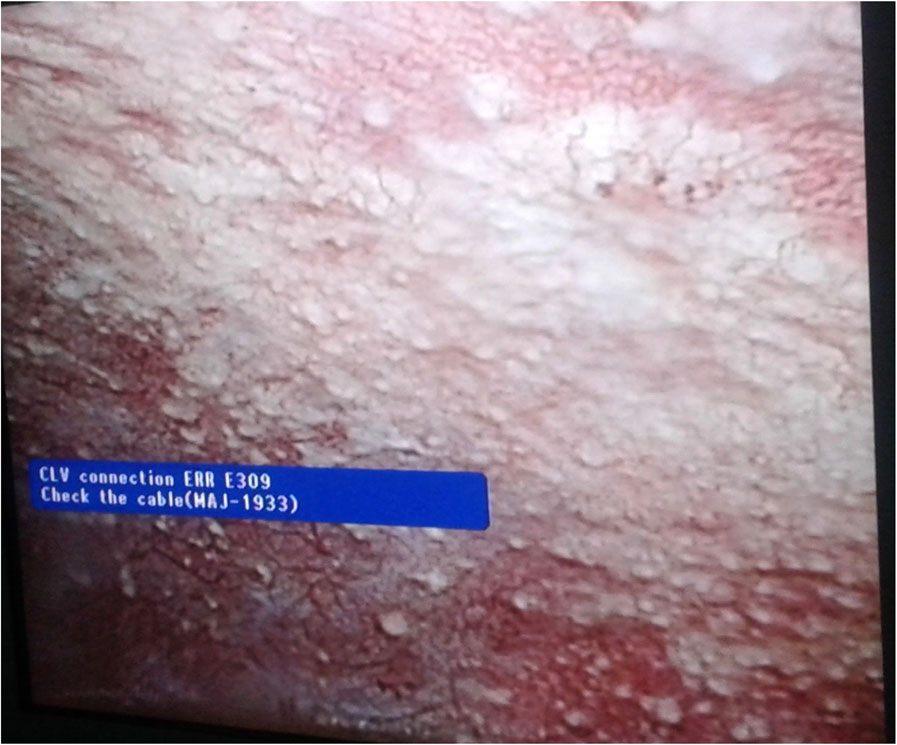
Peritoneal deposits visualized at laparoscopy

**Fig. 7:**
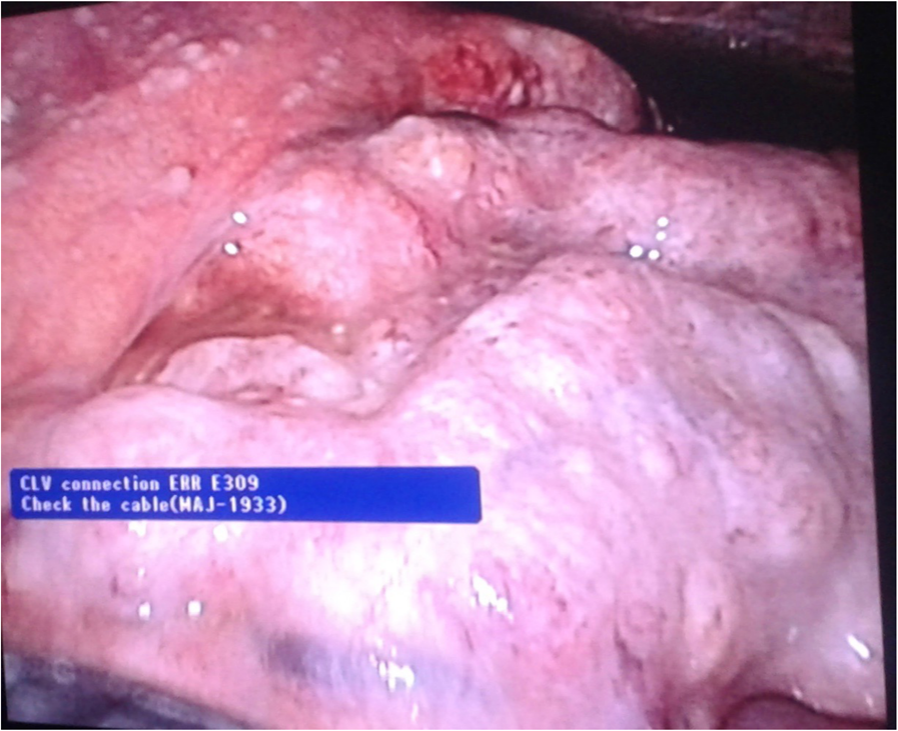
Omental deposits visualized at laparoscopy

**Fig. 8:**
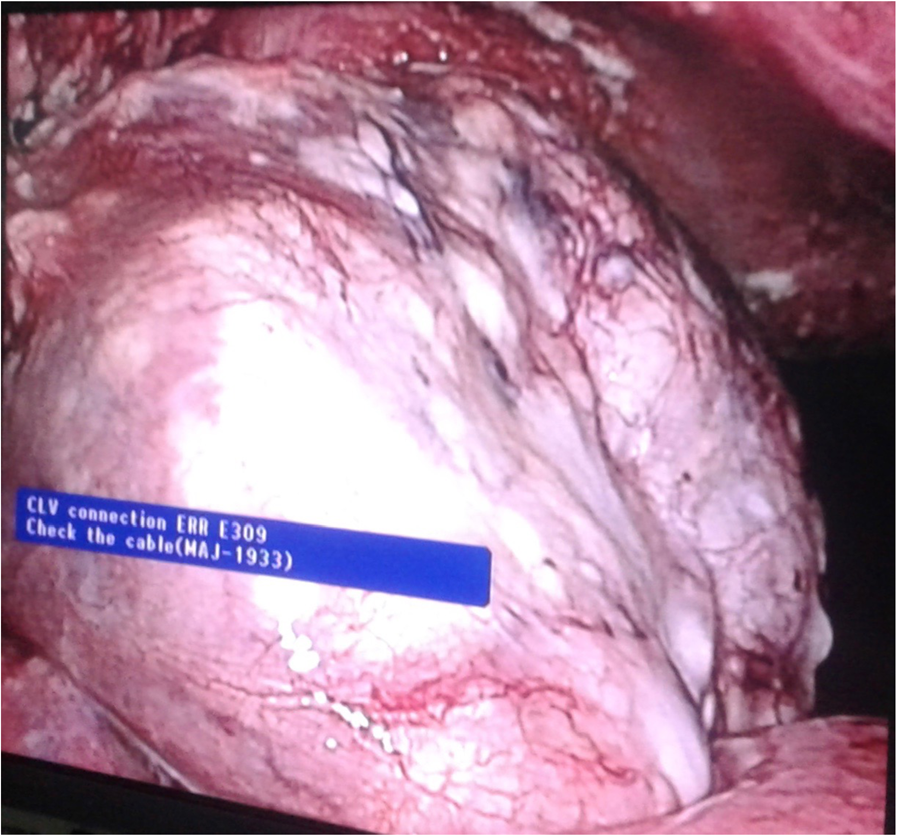
Liver lesion seen on the left lobe of the liver during laparoscopy

Laparoscopic assessment revealed gross ascites, peritoneal deposits (Fig. 6), and omental deposits (Fig. 7) with bowel adhesions. Ascitic fluid was drained and cytological analysis of 500 mL was negative for malignant cells. A liver lesion was seen at the left lobe of his liver (Fig. 8) and biopsies were taken from the liver lesion as well as omental and peritoneal deposits. TB culture, TB PCR, and GeneXpert for *Mycobacterium tuberculosis* in material retrieved from the liver lesion, omental deposit, and peritoneal deposit were negative. The biopsy from his liver showed nests and trabeculae of cells with vesicular nuclei and prominent nucleoli with sinusoidal pattern focally. Cytoplasmic bile staining and intranuclear inclusions were not seen. A biopsy from his peritoneum and omentum showed nests of similar cells infiltrating the desmoplastic stroma. These findings supported a diagnosis of HCC which was confirmed by immunohistochemistry.

He refused any surgical or oncological interventions and wished to be managed conservatively. He died 3 months later.

## Discussion

There is much research on the etiology and management of HCC in the cirrhotic liver. Data on the natural history of HCC in non-cirrhotic patients are limited. A variety of risk factors for the development of HCC have been identified including cirrhosis of almost any cause, mostly chronic viral hepatitis B or C, alcohol, nonalcoholic steatohepatitis, and hemochromatosis. HCC usually develops in the setting of chronic liver cirrhosis or inflammation [5]; less than 20% of HCCs develop in the non-cirrhotic liver [6]. As compared to HCC in a cirrhotic liver, HCC in a non-cirrhotic liver has some peculiarities, such as: lower male preponderance; bimodal age distribution; lower prevalence of the three main risk factors of hepatitis B virus (HBV) infection, HCV infection, and alcohol abuse; and increased prevalence of other etiologic factors (exposure to genotoxic substances and sex hormones, inherited diseases, and genetic mutations). Extrahepatic extension is also more common in HCC in patients who do not have cirrhosis [7, 8]. In the majority of patients with HCC in a non-cirrhotic liver, the etiology can most likely be related to metabolic syndrome [9]. It is also very rare to develop HCC in a normal liver without HBV and HCV infection.

One case report described a 36-year-old woman with normal liver function and negative hepatitis virus markers; abdominal USS, CT, and magnetic resonance imaging (MRI) showed a tumor in segment 7 of her liver. Upon aspiration biopsy of her liver, the histopathological diagnosis was moderately differentiated HCC [10]. Tanaka *et al*. [3] described a 39-year-old woman with normal liver function and no evidence of past and persistent HBV or HCV infection, whose abdominal ultrasonography and abdominal CT scan demonstrated a liver tumor of approximately 40 mm in maximal diameter in the lateral segment. This tumor was a moderately differentiated HCC. Tanaka *et al*. concluded that it is very rare to develop HCC in a normal liver without HBV and HCV infection [3]. Liver cancer is considered to be comparatively rare in young patients. According to the 16th report of the national follow-up study of primary liver cancer by Liver Cancer Study Group of Japan, only 0.6% of all cases of HCC occurred in patients younger than 35 years.

“Failure mode and effects analysis” [11] can be applied as a clinical reasoning tool to our patient. HCC is the potential failure. Effects of failure/main clinical features were progressive abdominal distension, discomfort, loss of appetite, and loss of weight [11]. There was no biochemical or radiological evidence of cirrhosis in our patient. We have excluded most of the risk factors for the development of HCC. The results of both the hepatitis B surface antigen and hepatitis B surface antibody tests were negative which excluded acute or chronic hepatitis B infection and previous exposure. This was also confirmed by negative hepatitis B core antibodies (IgM and IgG). Negative hepatitis C serology (anti-HCV antibody and hepatitis C RNA) excluded hepatitis C infection. He was abstinent from alcohol and did not have diabetes mellitus, hypertension, obesity, or hypertriglyceridemia suggesting metabolic syndrome, which is associated with nonalcoholic steatohepatitis. He did not have a history of exposure to aflatoxin or other environmental toxins, which can contribute to the pathogenesis of HCC. Normal iron studies and ceruloplasmin levels excluded hemochromatosis and Wilson disease. He did not have any features of autoimmune hepatitis and an ANA test was negative. His alpha-1 antitrypsin level was not tested due to financial constraints. However, he had no features of emphysema or panniculitis. Therefore we conclude that this is a rare case of a 41-year-old patient with HCC in a non-cirrhotic liver with negative hepatitis serology. Ascitic fluid was an exudate (serum-to-ascites albumin gradient was less than 11 g/L) and was lymphocyte predominant. His adenosine deaminase level, a purine-degrading enzyme needed for maturation and differentiation of lymphoid cells, can be used to detect tuberculous peritonitis and in this case was not supportive of TB peritonitis. TB PCR and TB culture were also negative. We sent approximately 700 mL of ascitic fluid for cytological analysis as the sensitivity increases up to 80 to 90% with increasing volume of ascitic fluid. However, atypical cells were not found.

HCC is highly aggressive and often detected at a late stage. As in our patient, the symptoms are not specific in the early stages of the disease. More than 60% of patients are diagnosed at a late stage of disease after metastasis has occurred [12]. Since early stage disease has a relatively good prognosis, it is important to identify the lesion early. Alpha fetoprotein is the most commonly used biomarker for HCC detection and some other new biomarkers such as glypican-3, osteopontin, Golgi protein 73, nucleic acids including microRNAs, Dickkopf-1 (DKK1), and midkine (MDK) will become clinically available in the near future [13, 14]. Chiou and Lee [15] identified two mediators of inflammation, S100 calcium-binding protein A9 (S100A9) and granulin protein markers, as HCC-associated cancer-specific biomarkers. They belong to the cytoplasmic alarmin family of the host innate immune system [15]. Even though these biomarkers were not available to us, in future they will be useful to identify HCC at an early stage.

The most frequent locations for metastasis of HCC were lungs, portal vein, bones, and regional lymph nodes [16–18]. Although the risk of metastasis in a ruptured HCC is high [4], intraperitoneal metastasis of a non-ruptured HCC is rare with an incidence of 2 to 6% detected during autopsy or laparoscopy [19]. Peritoneal metastases can occur synchronously or metachronously [20]. Our patient did not have a ruptured HCC. Sudden onset epigastric or right hypochondrial pain was the main reported complaint of patients with a ruptured HCC [21, 22]. Physical signs of hemodynamic instability, reduced hemoglobin level, and a raised aspartate aminotransferase level were more frequently found in patients with ruptured HCC than in those with non-ruptured tumors [22]. Peripheral location, protruding contour, discontinuity of hepatic surface, surrounding hematoma, and the “enucleation sign” are helpful signs for the diagnosis of a ruptured HCC in CT [23]. None of these were present in our patient.

Intraperitoneal seeding from a hepatoma can present as intraperitoneal masses, peritoneal thickening, and ascites. The main vascular feeders to intraperitoneal masses were omental branches of the gastroduodenal artery and/or the superior mesenteric artery [24]. The radiological manifestations of intraperitoneal metastasis from a HCC are single or multiple discrete hypervascular masses in the omentum. On angiography, the omental masses will show hypervascularity and are supplied by the omental branches of the superior mesenteric and inferior mesenteric arteries with prominent draining veins [25].

Surgery appears to be the optimal approach of managing extrahepatic metastasis of HCC localized to the peritoneum after considering the performance status, liver function, and tumor biology. The prognosis after cytoreductive surgery is likely to be related to the severity of chronic liver disease, the extent of peritoneal tumor involvement, and the response to surgery [26]. Resection of peritoneal metastases should be considered in patients whose primary liver neoplasm is under control and who have no metastases in other organs [20, 27]. Unfortunately, our patient decided to be managed conservatively without any interventions.

## Conclusions

HCC usually develops in the setting of chronic liver cirrhosis or inflammation associated with chronic hepatitis B and hepatitis C. Here we report a rare case of a large HCC in the non-cirrhotic liver of a 41-year-old man without chronic hepatitis presenting with peritoneal and omental metastasis. As compared to HCC in a cirrhotic liver, these tumors have some differences in epidemiology and etiology. Intraperitoneal metastasis of a non-ruptured HCC is very rare and cytoreductive surgery should be considered in those patients after careful assessment.

## Abbreviations

ANA: Antinuclear antibodies
CRP: C-reactive protein
CT: Computed tomography
CXR- PA: Posteroanterior chest X-ray
DKK1: Dickkopf-1
ESR: Erythrocyte sedimentation rate
HBV: Hepatitis B virus
HCC: Hepatocellular carcinoma
HCV: Hepatitis C virus
LDH: Lactate dehydrogenase
MDK: Midkine
PCR: Polymerase chain reaction
RBS: Random blood sugar
S100A9: S100 calcium-binding protein A9
TB: Tuberculosis
USS: Ultrasound
VDRL: Venereal Disease Research Laboratory
